# Loss of Weight Gained During the COVID-19 Pandemic: Content Analysis of YouTube Videos

**DOI:** 10.2196/35164

**Published:** 2022-02-09

**Authors:** Hao Tang, Sungwoo Kim, Priscila E Laforet, Naa-Solo Tettey, Corey H Basch

**Affiliations:** 1 Department of Health and Behavior Studies Teachers College Columbia University New York, NY United States; 2 Department of Human Development Teachers College Columbia University New York, NY United States; 3 Mailman School of Public Health Columbia University New York, NY United States; 4 Department of Public Health William Paterson University Wayne, NJ United States

**Keywords:** COVID-19, quarantine, weight loss, weight gain, social media, YouTube

## Abstract

**Background:**

Many people experienced unintended weight gain during the COVID-19 pandemic, which has been discussed widely on social media.

**Objective:**

This study aims to describe the content of weight loss videos on YouTube (Google LLC) during the COVID-19 pandemic.

**Methods:**

By using the keywords *weight loss during quarantine*, the 100 most viewed English-language videos were identified and coded for content related to losing weight gained during the COVID-19 pandemic.

**Results:**

In total, 9 videos were excluded due to having non-English content or posting data before the COVID-19 pandemic. The 91 videos included in the study sample acquired 407,326 views at the time of study and were roughly 14 minutes long. A total of 48% (44/91) of the sample videos included graphic comparisons to illustrate weight change. Videos that included a graphic comparison were more likely to have content related to trigger warnings (*χ*^2^_1_=6.05; *P*=.01), weight loss (*χ*^2^_1_=13.39; *P*<.001), negative feelings during quarantine (*χ*^2^_1_=4.75; *P*=.03), instructions for losing weight (*χ*^2^_1_=9.17; *P*=.002), self-love (*χ*^2^_1_=6.01; *P*=.01), body shaming (*χ*^2^_1_=4.36; *P*=.04), and special dietary practices (*χ*^2^_1_=11.10; *P*<.001) but were less likely to include food recipes (*χ*^2^_1_=5.05; *P*=.03). Our regression analysis results suggested that mentioning quarantine (*P*=.05), fat-gaining food (*P*=.04), self-care and self-love (*P*=.05), and body shaming (*P*=.008) and having presenters from both sexes (*P*<.001) are significant predictors for a higher number of views. Our adjusted regression model suggested that videos with content about routine change have significantly lower view counts (*P*=.03) than those of videos without such content.

**Conclusions:**

The findings of this study indicate the ways in which YouTube is being used to showcase COVID-19–related weight loss in a pre-post fashion. The use of graphic comparisons garnered a great deal of attention. Additional studies are needed to understand the role of graphic comparisons in social media posts. Further studies that focus on people’s attitudes and behaviors toward weight change during the COVID-19 pandemic and the implications of social media on these attitudes and behaviors are warranted.

## Introduction

COVID-19 is a contagious respiratory illness caused by the novel coronavirus (SARS-CoV-2). The World Health Organization declared the COVID-19 outbreak a global pandemic due to its rapid spread and alarming severity in March 2020 [1]. In an early attempt to reduce the transmission of SARS-CoV-2 (ie, to “flatten the curve”) [2], over 100 countries have implemented self-quarantine at different points since January 2020 (referred to as *lockdown*) [2,3]. In March 2020, different regions in the United States enforced lockdowns of varying stringency, with most regions restricting outdoor activities and shutting down schools and other nonessential businesses [4,5]. Some states announced stay-at-home orders with a mandate that all nonessential work was to be conducted from home [4]. After the COVID-19 vaccines became available to the general public in December 2020, businesses gradually reopened, but social distancing was largely still encouraged [6]. The unprecedented shutdown has effectively slowed the spread of COVID-19 and averted an estimated 531 million coronavirus infections around the world, including 60 million infections in the United States [7]. Despite this however, the negative consequences of the COVID-19 pandemic persist. These include job losses [8] and the increased prevalence of mental health issues (eg, depression and anxiety) [9,10], as well as a myriad of additional societal losses [11]. The COVID-19 quarantine has also resulted in significant lifestyle changes, such as variations to customary eating habits and physical activity [12,13]. These changes have resulted in widespread concerns about weight gain and body image [14]. Reflecting this, the term *quarantine 15* is highly discussed on social media [15,16]. For example, a recent search yielded approximately 50,600 videos on YouTube (Google LLC) that include *quarantine 15* in their titles. Further, a cursory search of the term *quarantine 15* yielded more than 619,000 Instagram posts, not including thousands of posts using related terms and those found in other social media outlets, such as Twitter and Facebook.

Social media has long been a popular tool for sharing and disseminating prompt, health-related information and a cost-effective information and education platform that can help with intervening in health behaviors [17], including weight management. For example, weight loss is one of the most searched topics on the internet [17]. However, not all social media use is positive, especially when it comes to promoting health behaviors such as safe and effective weight loss. For example, content that stigmatizes weight gain or triggers eating disorders is present on social media [16,18]. Moreover, recent evidence suggests that image-centric social media platforms have a greater impact on body image dissatisfaction and eating disorder behaviors than non–image-centric social media platforms [19]. As one of the most popular video-sharing platforms around the globe [20-22], YouTube has been the focal point of a range of studies related to COVID-19. Given the widespread concerns about undesired weight gain, the popularity of YouTube, and the long-existing risk of social media content [14,16,17], it is important to characterize the trending weight-related social media posts during the COVID-19 quarantine to investigate the effect of quarantine on individuals’ behaviors and health [16]. Therefore, the purpose of this study was to describe the content of weight loss videos on YouTube during the COVID-19 pandemic.

## Methods

This was a qualitative, content analysis study that adapted methods from prior studies that analyzed YouTube videos on COVID-19 vaccination [23,24]. By using “visitor” mode, we conducted searches with the keywords *quarantine weight loss* and *quarantine weight gain* and found that the results were similar. To keep the view count as a valid measure, we used 1 key term—*quarantine weight loss*—to filter the first 100 videos by view count. However, 6 of the most viewed videos were not presented in English, and 3 videos were posted before the pandemic (ie, before 2019). As such, only 91 videos were coded and analyzed.

A total of 23 coding categories were used to code each video, and only 7 basic information categories [25], including (1) URL, (2) upload date, (3) view count, (4) thumbs-up, (5) thumbs-down, (6) video length (in minutes and seconds), and (7) presenter sex, were coded and recorded on the same day. Thumbs-up and thumbs-down counts were subsequently used to calculate the like to dislike ratio. Additional categories were extracted from related articles, a World Health Organization report, and the first 10 most viewed videos [14,25-27]. These characteristic categories were (1) including a trigger warning or disclaimer, (2) mentioning quarantine, (3) including a graphic comparison of pre- and post-pandemic weight (cover, picture, or video), (4) mentioning weight gain during quarantine, (5) mentioning an exact amount of quarantine weight gain, (6) mentioning weight loss during quarantine, (7) mentioning an exact amount of quarantine weight loss, (8) mentioning personal causes of quarantine weight gain, (9) mentioning negative feelings during quarantine, (10) highlighting food of low nutritional quality (dessert or ultraprocessed food), (11) mentioning exercise, (12) mentioning how to lose weight during quarantine, (13) highlighting weight loss pills or products, (14) mentioning self-love or self-care, (15) mentioning body shaming, (16) mentioning a specific diet, and (17) including recipes.

Each video was coded as “1” (yes) or “0” (no) for whether the video mentioned these characteristic categories. Means, SDs, and ranges were calculated for the view count, thumbs-up, thumbs-down, and video length variables. Frequencies and percentages were calculated for all categorical variables. Chi-square tests were used to investigate associations between the inclusion of a graphic comparison of pre- and postpandemic weight and other video characteristics. In addition, a correlation analysis was conducted to determine if there were significant relationships among various video characteristics. Finally, a regression analysis was conducted, with view count (in thousands) as a dependent variable and different video characteristics as independent variables, to test for significant predictors of higher view counts. After the initial interpretation of results, a stepwise regression was performed to reduce the complexity of our model and produce a more efficient model. The descriptive analyses (correlation and chi-square tests) were performed by using SPSS version 27 (IBM Corporation), and the regression analysis with subset selection was conducted with RStudio 1.4.1717. This study was not reviewed by the institutional review boards of Columbia University and William Paterson University because it did not involve human subjects, per their policies.

## Results

The 91 YouTube videos on weight loss during quarantine had an average of 407,326 views; this value had a high SD of 836,478 views. Most (65/91, 71%) of the videos were uploaded between January 1 and August 2, 2020, and only 29% (26/91) of videos were uploaded between August 2, 2020, and March 2, 2021. The majority of videos were presented by females (65/91, 71%). The videos were roughly 14 minutes long on average, though the length ranged from 2 minutes to 1 hour. Most videos were very positively rated; the average like to dislike ratio was 98%. A complete list of the characteristics included in these videos is outlined in Table 1.

**Table 1:** Characteristics of the most viewed YouTube videos on quarantine weight loss (N=91).

Characteristics		Values
**Upload date of videos, n (%)**		
	Between January 1 and August 2, 2020	65 (71)
	Between August 2, 2020, and March 2, 2021	26 (29)
View count, mean (SD; range)		407,326,69 (836,478.02; 44,181-5,396,499)
Thumbs-up (like) count, mean (SD; range)		13,004.82 (24,237.7; 639-176,000)
Thumbs-down (dislike) count, mean (SD; range)		203.31 (297.4; 16-1500)
Video length (seconds), mean (SD; range)		847.19 (614.8; 141-4063)
**Presenter sex, n (%)**		
	Female	65 (71)
	Male	22 (24)
	Both	4 (4)
**Includes a trigger warning or disclaimer, n (%)**		
	Yes	14 (15)
	No	77 (85)
**Mentions quarantine, n (%)**		
	Yes	83 (91)
	No	8 (9)
**Includes a graphic comparison of pre- and postpandemic weight (cover, picture, or video), n (%)**		
	Yes	44 (48)
	No	47 (52)
**Mentions the exact amount of quarantine weight gain, n (%)**		
	Yes	16 (18)
	No	75 (82)
**Mentions the exact amount of quarantine weight loss, n (%)**		
	Yes	37 (42)
	No	52 (58)
**Mentions weight gain during quarantine, n (%)**		
	Yes	29 (32)
	No	62 (68)
**Mentions weight loss during quarantine, n (%)**		
	Yes	57 (63)
	No	34 (37)
**Mentions personal causes of quarantine weight gain, n (%)**		
	Yes	17 (19)
	No	74 (81)
**Mentions negative feelings during quarantine, n (%)**		
	Yes	39 (43)
	No	52 (57)
**Highlights fat-gaining food (dessert or ultraprocessed food), n (%)**		
	Yes	17 (19)
	No	74 (81)
**Mentions exercise, n (%)**		
	Yes	62 (68)
	No	29 (32)
**Mentions how to lose weight during quarantine, n (%)**		
	Yes	38 (42)
	No	53 (58)
**Highlights weight loss pills or products, n (%)**		
	Yes	24 (26)
	No	67 (74)
**Mentions self-love or self-care, n (%)**		
	Yes	30 (33)
	No	61 (67)
**Mentions body shaming, n (%)**		
	Yes	10 (11)
	No	81 (89)
**Mentions a specific diet, n (%)**		
	Yes	27 (30)
	No	64 (70)
**Includes recipes, n (%)**		
	Yes	33 (36)
	No	58 (64)

Table 2 features a compilation of chi-square tests of independence for testing the relationship between the inclusion of a graphic pre-post weight comparison and various other video characteristics. Videos that included a trigger warning were more likely to feature a graphic comparison (*χ*^2^_1_=6.05; *P*=.01). Such videos that included a graphic comparison of pre- and postpandemic weight also mentioned weight loss more often than videos without a graphic comparison (*χ*^2^_1_=13.39; *P*<.001) and more often mentioned negative feelings during quarantine (*χ*^2^_1_=4.75; *P*=.03). In addition, videos with a graphic comparison more frequently included how-to instructions (*χ*^2^_1_=9.17; *P*=.002) and more frequently mentioned self-love (*χ*^2^_1_=6.01; *P*=.01), body shaming (*χ*^2^_1_=4.36; *P*=.04), and special dietary practices (*χ*^2^_1_=11.10; *P*<.001). However, videos with graphic comparisons significantly less often included food recipes (*χ*^2^_1_=5.05; *P*=.03).

**Table 2:** Associations between the inclusion of a graphic comparison of pre- and postpandemic weight and video characteristics.

Categories			Includes a graphic comparison							Chi-square (*df*)			*P* value
			No, n		Yes, n		Total, N						
**Presenter sex**			47		44		91		0.47 (2)			.79	
	Female	35		30		65							
	Male	10		12		22							
	Both	2		2		4							
**Includes a trigger warning or disclaimer**			47		44		91		6.05 (1)			.01^a^	
	No	44		33		77							
	Yes	3		11		14							
**Mentions quarantine**			47		44		91		0.01 (1)			.92	
	No	4		4		8							
	Yes	43		40		83							
**Mentions weight gain**			47		44		91		0.79 (1)			.37	
	No	34		28		62							
	Yes	13		16		29							
**Mentions weight loss**			47		44		91		13.39 (1)			<.001^a^	
	No	26		8		34							
	Yes	21		36		57							
**Includes a COVID-19 weight change–related term**			47		43		90		9.56 (1)			.002^a^	
	No	45		31		76							
	Yes	2		12		14							
**Mentions weight gain cause**			47		44		91		0.92 (1)			.34	
	No	40		34		74							
	Yes	7		10		17							
**Mentions negative feelings during quarantine**			47		44		91		4.75 (1)			.03^a^	
	No	32		20		52							
	Yes	15		24		39							
**Mentions fat-gaining food**			47		44		91		2.24 (1)			.14	
	No	41		33		74							
	Yes	6		11		17							
**Mentions exercise**			47		44		91		3.28 (1）			.07	
	No	19		10		29							
	Yes	28		34		62							
**Mentions routine or life change**			47		44		91		0.07 (1)			.79	
	No	19		19		38							
	Yes	28		25		53							
**Mentions how to lose weight**			47		44		91		9.17 (1)			.002^a^	
	No	34		18		52							
	Yes	13		26		39							
**Highlights weight loss pills or products**			47		44		91		0.04 (1)			.85	
	No	35		32		67							
	Yes	12		12		24							
**Mentions self-love or self-care**			47		44		91		6.01 (1)			.01^a^	
	No	37		24		61							
	Yes	10		20		30							
**Mentions body shaming**			47		44		91		4.51 (1)			.03^a^	
	No	45		36		81							
	Yes	2		8		10							
**Mentions a specific diet (eg, keto diet, etc)**			47		44		91		10.17 (1)			.001^a^	
	No	40		24		64							
	Yes	7		20		27							
**Includes recipes**			47		44		91		3.68 (1)			.03^a^	
	No	25		33		58							
	Yes	22		11		33							

^a^Statistically significant at a *P*<.05 significance level.

A regression analysis with a full list of 21 factors was conducted to create a statistically significant model (*F*_21,68_=3.223; *P*<.001) with moderate model fit (*R*^2^=34.4%). Videos that mentioned COVID-19 quarantine had approximately 580,000 more views than those that did not mention COVID-19 quarantine (*P*=.05). In addition, talking about fat-gaining food (*P*=.04), self-love (*P*=.05), and body shaming (*P*=.008) significantly contributed to higher view counts. Further, videos with both male and female presenters had, on average, 1.8 million more views than videos with male presenters and 1.5 million more views compared to videos with female presenters (*P*<.001). Table 3 outlines the full list of regression coefficients.

**Table 3:** Regression analysis with full factors.

Characteristics	B (SE)	*t* test (*df*)^a^	*P* value
Intercept	−3420.035 (2411.484)	−1.418 (68)	.16
Like to dislike ratio	3537.054 (2449.459)	1.444 (68)	.15
Length (seconds)	−0.241 (0.141)	−1.714 (68)	.09
Includes a trigger warning	278.817 (236.567)	1.179 (68)	.24
Mentions quarantine	580.534 (290.706)	1.997 (68)	.05^b^
Mentions a graphic comparison	−17.898 (205.592)	−0.087 (68)	.93
Mentions weight gain	−111.256 (205.264)	−0.542 (68)	.59
Mentions weight loss	−295.420 (174.849)	−1.690 (68)	.10
Includes a COVID-19 weight change–related term	−217.298 (229.365)	−0.947 (68)	.35
Mentions weight gain cause	−535.506 (264.648)	−2.023 (68)	.047^b^
Mentions negative feelings	128.832 (188.775)	0.682 (68)	.50
Mentions fat-gaining food	470.720 (218.611)	2.153 (68)	.04^b^
Mentions exercise	28.272 (176.027)	0.161 (68)	.87
Mentions a routine or life change	−392.194 (209.015)	−1.876 (68)	.07
Mentions how to lose weight	350.152 (182.994)	1.913 (68)	.06
Highlights weight loss pills or products	16.195 (190.823)	0.085 (68)	.93
Mentions self-love or self-care	389.582 (195.430)	1.993 (68)	.05^b^
Mentions body shaming	700.194 (255.749)	2.738 (68)	.008^c^
Mentions a specific diet (eg, keto, etc)	−56.951 (198.153)	−0.287 (68)	.78
Includes recipes	−113.440 (179.326)	−0.633 (68)	.53
Presenter is female	269.498 (187.232)	1.439 (68)	.16
Includes both male and female presenters	1848.469 (394.523)	4.685 (68)	<.001^c^

^a^Values are from a regression analysis.

^b^Statistically significant at a *P*<.05 level.

^c^Statistically significant at a *P*<.01 level.

To reduce the number of parameters and improve model fit, forward and backward stepwise regressions were performed. Among the two models, the backward stepwise regression model was selected because it had a lower Akaike Information Criteria value (1195.92) compared to that of the forward regression model (1208.15). The selected model was, overall, significantly similar to the full model (*F*_11,79_=5.506; *P*<.001) and had a slightly improved model fit (*R*^2^=35.5%). The reduced model included 11 out of the 21 variables, and the complete list of the coefficients can be found in Table 4. The effects of mentioning quarantine (*P*=.049) and body shaming (*P*=.009) and presenter sex (*P*<.001) stayed significant. In addition, mentioning changes in life routine was selected as a significant predictor of view count (*P*=.03), though videos that included content about routine change had approximately 380,000 fewer views.

**Table 4:** Backward stepwise regression analysis with reduced variables^a^.

Characteristics	B (SE)	*t* test (*df*)^b^	*P* value
Intercept	−3323.7 (2291.4)	−1.451 (79)	.15
Like to dislike ratio	3329.7 (2304.5)	1.445 (79)	.15
Length (seconds)	−0.2 (0.1)	−1.778 (79)	.08
Mentions quarantine	556.1 (278.4)	1.997 (79)	.049^c^
Mentions weight gain	−271.6 (167.1)	−1.626 (79)	.11
Highlights fat-gaining food	342.1 (192.4)	1.778 (79)	.08
Mentions a routine or life change	−385.6 (173.4)	−2.212 (79)	.03^c^
Mentions how to lose weight	296.8 (157.1)	1.889 (79)	.06
Mentions self-love or self-care	221.5 (165.5)	1.339 (79)	.18
Mentions body shaming	630.9 (237.1)	2.660 (79)	.009^d^
Presenter is female	246.0 (171.9)	1.432 (79)	.16
Includes both male and female presenters	1889.4 (369.1)	5.100 (79)	<.001^d^

^a^The Akaike Information Criteria and Bayesian Information Criteria values of the model were 1195.92 and 1195.92, respectively.

^b^Values are from a regression analysis.

^c^Statistically significant at a *P*<.05 level.

^d^Statistically significant at a *P*<.01 level.

## Discussion

The findings of this study are important in that they indicate the ways in which YouTube is being used to showcase weight loss in a pre-post fashion. Further, videos that used graphic comparisons garnered the most attention and included less of the studied content compared to those that did not use such comparisons. The power of graphic depiction has long been recognized in many fields, including research, education, and business [28-30]. One example is the social comparison theory, which posits that self-worth is often determined through the assessment of differences and similarities with others [31]. This highlights why exposure to body images on social media can result in both positive and negative consequences [32,33].

Many studies have found that social media posts with pictures usually induce higher levels of engagement with eating disorder behaviors than those induced by posts without pictures [34,35]. In this study, videos that included a graphic comparison of pre- and postpandemic weight usually talked about weight loss (mentions weight loss, mentions how to lose weight, and mentions the exact amount of quarantine weight loss) but not weight gain. It is possible that weight loss has always been a popular topic in social media [36]. As such, video makers wanted to make their video content stand out by showing the efficacy of their weight loss journeys with graphic comparisons.

On the other hand, it should be noted that graphic posts can magnify the risk of social media use with regard to body objectification, body dissatisfaction, and eating disorders [19,37-40]. Graphic comparison is a strategy that is used to motivate participants by demonstrating the potential results of following a suggested regimen or advice. However, instead of promoting body positivity, some graphic comparisons have the opposite effect because they are based on the erroneous belief that fat shaming or weight stigma can serve as a motivator for weight loss [41,42]. Transformational graphic images often focus on decreased size and high amounts of weight loss as determinants of good health. The linking of weight to health can lead to negative body image and decreased self-esteem for those who do not meet the criteria in the posted images or videos [43]. In addition, this strategy does not often lead to motivation but instead can discourage and decrease the self-efficacy of viewers who do not believe that they can achieve the weight loss goals portrayed in such images or videos [43]. Furthermore, considering racial and ethnic representation and cultural body image standards within these images is important for reaching a diverse audience and achieving body inclusivity [44].

Including trigger warnings could be an effective strategy for limiting exposure to content that can distort body image. We found that videos that include a pre-post graphic comparison are more likely to include trigger warnings or disclaimers and mention self-love, self-care, and body shaming compared to those without a graphic comparison. Only 15% (14/91) of these popular videos discussing weight change included trigger warnings or disclaimers. Social media platforms and content creators should be more aware of the potential risk of content related to body image and promote policies to reduce this risk. Future studies should seek to develop best practices for developing graphic images in a way that promotes health and body positivity instead of just weight loss and a thin body ideal.

The literature indicates that several lifestyle changes during the COVID-19 quarantine have resulted in weight gain and increased the risk for obesity [45,46], which is a primary public health concern [47,48]. Studies have confirmed that weight gain was commonplace around the globe during the COVID-19 quarantine [14,45,46,49]. The interruption of usual routines and restriction of social behaviors due to the COVID-19 quarantine can result in increased boredom [12] and stress [50], which in turn induce emotional eating and food craving [51-53]*.* Additionally, higher energy intake; the higher consumption of sugar, fat, and alcohol; and the limited availability of fresh fruits and vegetables [13,54] during quarantine increased the risk for overweight and many metabolic diseases [55]. In addition, the decrease in physical activity and increase in sedentary behaviors, such as screen time, during the COVID-19 quarantine may also contribute to weight and fat gain [56].

This study is limited by its cross-sectional design, the sole inclusion of English-language videos, and the search term being limited to 1 phrase. There is no indication of how our results may have differed at other points in time during the pandemic, as quarantine rules varied greatly over time. Despite these limitations, this study does contribute to a gap in the literature and may encourage researchers to conduct studies related to the loss of weight gained during the COVID-19 pandemic.

Social media use increased significantly during the COVID-19 pandemic, since it has helped people feel connected with others and has kept them updated with news and entertained while staying at home [16,57]. More than half of US adults reported the increased use of social media platforms after the pandemic [58]. Social media sites like YouTube provide an opportunity for lay content creators, as well as public health organizations, to reach large audiences and provide content that can promote improved body image and increased focus on health rather than weight [59]. The lessons learned from the COVID-19 pandemic should serve as a catalyst for public health practitioners to develop evidence-based tools that people can use to remain healthy should an extended quarantine occur again. Many creators of social media content that focuses on weight loss are not trained in health education or public health and may not be using evidence-based strategies to develop content. It would be beneficial for trusted organizations to develop evidence-based social media education and training with guidelines for maintaining a healthy weight and establishing healthy behaviors during times of quarantine or other public health emergencies. Further studies that focus on people’s attitudes and behaviors toward weight change during the COVID-19 pandemic and the implications of social media on these attitudes and behaviors are warranted.
